# Proposed mechanism for the length dependence of the force developed in maximally activated muscles

**DOI:** 10.1038/s41598-018-36706-4

**Published:** 2019-02-04

**Authors:** Lorenzo Marcucci, Takumi Washio, Toshio Yanagida

**Affiliations:** 1 Department of Biomedical Sciences, Padova University, Via Marzolo 3, 35131 Padova, Italy; 2 Center for Mechanics of Biological Materials, Padova University, Via Marzolo 9, 35131 Padova, Italy; 3 Center for Biosystems Dynamics Research, RIKEN, 6-2-3 Furuedai, Suita, Osaka 565-0874 Japan; 4 Graduate School of Frontier Sciences, The University of Tokyo, 5-1-5 Kashiwanoha, Kashiwa-shi, Chiba-ken 277-8561 Japan

## Abstract

The molecular bases of the Frank-Starling law of the heart and of its cellular counterpart, the length dependent activation (LDA), are largely unknown. However, the recent discovery of the thick filament activation, a second pathway beside the well-known calcium mediated thin filament activation, is promising for elucidating these mechanisms. The thick filament activation is mediated by the tension acting on it through the mechano-sensing (MS) mechanism and can be related to the LDA via the titin passive tension. Here, we propose a mechanism to explain the higher maximum tension at longer sarcomere lengths generated by a maximally activated muscle and test it *in-silico* with a single fiber and a ventricle model. The active tension distribution along the thick filament generates a *reservoir* of inactive motors at its free-end that can be activated by passive tension on a beat-to-beat timescale. The proposed mechanism is able to quantitatively account for the observed increment in tension at the fiber level, however, the ventricle model suggests that this component of the LDA is not crucial in physiological conditions.

## Introduction

Heart contraction provides blood supply to muscle and organs in our body and must adapt to external conditions that can change in the time scale of a single beat. Its ability to regulate the ejection volume of each beat to the end diastolic volume or preload, has been discovered respectively by Frank^1^ and Starling^2^ more than a century ago, and is known as the Frank-Starling law of the heart. However, the molecular bases of this mechanism are still under debate, partially because multiple mechanisms are behind this fine regulatory system. Shedding light on these mechanisms is important because an impaired ability of the heart to fulfill the Frank-Starling law is associated with hypertrophic cardiomyopathies^3^.

At the cellular level the Frank-Starling relation is reflected in the so-called length dependent activation (LDA), associated with a higher tension generated during the contraction at longer sarcomere lengths (SL). This can be seen in intact cardiac muscle cells with higher peak forces during twitches^4^, or in skinned muscle through the tension-pCa curve^5,6^. The sigmoidal tension-pCa curve is characterized by three components, as shown by its mathematical description through the Hill equation:

1 $$T([C{a}^{2+}])={T}_{max}\frac{{[C{a}^{2+}]}^{{n}_{H}}}{EC{{a}_{50}}^{{n}_{H}}+{[C{a}^{2+}]}^{{n}_{H}}},$$

the steepness, defined by the Hill parameter (n_H_), the maximum tension at high level of [Ca^2+^] (T_max_), and the [Ca^2+^] at which a half of T_max_ is generated, which indicates the sensitivity to calcium ions at intermediate [Ca^2+^] (ECa_50_ or the inverse of its logarithm pCa_50_). Whilst these three parameters must have some interrelationship, a certain degree of independency is shown experimentally, in particular on SL dependence which is statistically not significant (p = 0.2) for n_H_, while it is significant for both pCa_50_ and T_max_ (p < 0.001)^5^. Moreover, pCa_50_ and T_max_ represent two distinct components for the LDA, based on two different mechanisms: the first component, the increase of pCa_50_ at longer SLs, is largely explained by the activation of the thin filament, while the second component, the increase of T_max_, seems to be related to the activation of the thick filament^7^.

The two filaments activation system is a recent discovery in muscle field. Textbooks explain muscle force generation through the classical cross-bridge cycle, where a single myosin motor, starting from a detached state, protrudes from the thick filament and attach to the actin thin filament, forming a cross-bridge and exerting a force with a rotation of its lever-arm^8–10^. The thin filament is available for this interaction only when it is activated by calcium ions. Calcium ions are released by the action potential from the sarcoplasmic reticulum to the cytosolic space and bound to the troponin, shift the tropomyosin which runs along the thin filament and make the myosin-binding sites available for the cross-bridge cycle. Then, the [Ca^2+^] regulates the activation of the thin filament and the number of myosin motors which will attach to it. However, recent discoveries are evolving this well-established theory. When a muscle is not activated, myosin motors are in a super-relaxed, or OFF, state^11^, a low ATP consuming configuration where motors are packaged along the thick filament interacting with each other through intermolecular and intramolecular protein-protein interactions^12,13^. Myosin motors in the SRX state are not allowed to interact with the thin filament, even when it is activated. In other words, to generate force even the thick filament must be activated, rising the motors toward the thin filament in a disordered detached, or ON, state. Only in this situation they can attach with a probability which depends on the [Ca^2+^] and the thin filament activation. These two activations, which finely regulate muscle force, are not independent each other, because of the so-called mechanosensing (MS) mechanism. The MS mechanism relates the tension borne by the thick filament to the stability of the SRX motors: the higher is the tension, the higher the probability of activation of the single motor. It has a cooperative effect because a higher amount of active motors generates a higher amount of cross-bridge cycles and consequently a higher force sustained by the thick filament. MS mechanism has been discovered for the first time in the skeletal muscle^14^ and later observed also in cardiac muscle^15^, and influences the contraction in physiological conditions^16,17^, giving a feedback to each muscle cell to sense how much force is needed, leading, for instance, to a minimum number of working motors during unloaded contractions.

Here we explore the ability of the MS mechanism to generate a feedback of the SL through the passive tension generated by titin. In fact, MS mechanism is sensitive to both active and passive tensions^7,18^ suggesting that the LDA could be generated by an extra amount of motors activated by the titin passive tension, especially in the cardiac cells where titin is stiffer than skeletal isoforms^19^. In particular, we use a mathematical model to explain the higher T_max_ at longer SLs through the MS mechanism. We start form our previous model, which included a simplified description of the MS mechanism where the probability for each motor to be activated was affected by the total tension sustained by the thick filament, regardless of the position of the motor itself. That model reproduces the higher calcium sensitivity at intermediate [Ca^2+^], but fails to match the second feature of the force-pCa experimental curve, predicting the same T_max_ at any SL. Here we modify our previous model considering a different activation rate for each myosin motor, depending on the tension acting at its position. While passive tension is generated by titin before the free end of the thick filament, and is probably constant throughout its length, the active tension in each point is the sum of all the attached motors from there to the free end and increases toward the M-line. We show that the relatively low passive tension can have an important role, even at high [Ca^2+^], in the activation of the thick filament around its free-end because in that region the active tension is always low. We show that the proposed mechanism can quantitatively explain the higher T_max_ associated with longer SLs, generating a non-uniform distribution of the active motors along the thick filament. Notably, including the fiber model into a finite element (FE) left ventricle model, the new mechanism failed to improve the Frank-Starling relationship, opening the question about the relative importance of this component of the LDA in physiological situations.

## Results

### Model description and proposed mechanism

We used a Monte-Carlo approach to simulate the tension distribution along the thick filament and its influence on the state of each myosin motor in each filament of a half-sarcomere model. This model simulates the behavior of a single fiber under the assumption that all the half-sarcomeres contract uniformly. Under similar assumption the model is used to simulate a trabecula, assuming that the fibers occupy one-half of the whole cross-sectional area. Based on experimental evidences, myosin motors are modeled to possibly be in: (i) an ordered detached OFF state, (ii) a disordered detached ON state, or (iii) in an attached state with one pre power stroke and two-steps post power stroke states. Cross-bridge cycle is then divided into two main cycles as previously proposed^20^: a “resting cycle” between the OFF and ON states, and a “working cycle” between the ON and the attached states. The MS mechanism is introduced as a tension dependence of the rate function for the activation of the detached OFF myosin motors: k_OFF-ON_(T). Similarly to our previous model^20^, based on experimental data present in literature, k_OFF-ON_ has a small constant value ON_min_ up to a first threshold in tension T_th1_, then it increases linearly up to a higher constant value ON_max_ at a second threshold T_th2_. ON_min_ defines the constitutively ON myosin in the relaxed muscle, while ON_max_ defines the maximum activation which in our case is almost complete. Except where explicitly mentioned, the constitutively ON motors below T_th1_ are assumed to be randomly and uniformly distributed along the thick filament. All the figures in the text are based on this hypothesis, but a modification is introduced to simulate the stabilizing effect of the myosin binding protein C, and results are shown in the Supplementary material.

Passive force is uniformly distributed along total thick filament length (L_Thick_) and increases with the square of ΔSL = SL-1.9 μm^19^, while active force is the sum of the forces generated by each myosin attached to actin filament and increases along the thick filament from the free end toward the M-line. Active force generated by attached motors depends on their position into a three-minima energy landscape (related to the pre-power stroke and two post-power stroke states), which accounts for the thermal fluctuations of myosin heads, as previously described^21,22^. The parameters for the double calcium-binding site on each troponin-tropomyosin regulatory units (RU), are defined by the experimental data present in literature^7^ obtained through cardiac troponin with bifunctional rhodamine incorporated in rat trabeculae. Our model matches the value of the Hill parameter for the thin filament activation-pCa curve at SL = 1.9 µm (see Table 1), and its full activation at high [Ca^2+^] regardless the SL. To focus our attention on the thick filament activation, we did not include the observed thin filament activation dependence on SL, as well as the cross-bridge-RU cooperativity (see also Materials and Methods), to exclude any other role on T_max_ at longer SL.

**Table 1:** Fitting parameters for the Hill curves at different SLs and 95% confidence bounds.

SLs (µm)	ECa_50_ (µM)	T_max_ (kPa)	n_H_
1.9	1.24 (1.22, 1.26)	104.5 (103.8, 105.3)	3.53 (3.37, 3.69)
2.0	1.19 (1.17, 1.21)	108.8 (108.1, 109.6)	3.54 (3.39, 3.69)
2.1	1.13 (1.11, 1.15)	122.2 (121.3, 123.1)	3.42 (3.26, 3.58)
2.2	0.98 (0.97, 0.99)	140.6 (139.8, 141.4)	3.46 (3.32, 3.60)
2.3	0.88 (0.87, 0.89)	147.4 (146.8, 148.0)	2.95 (2.88, 3.02)

### Thick filament is neither fully nor uniformly activated at short SLs

**Figure 1:**
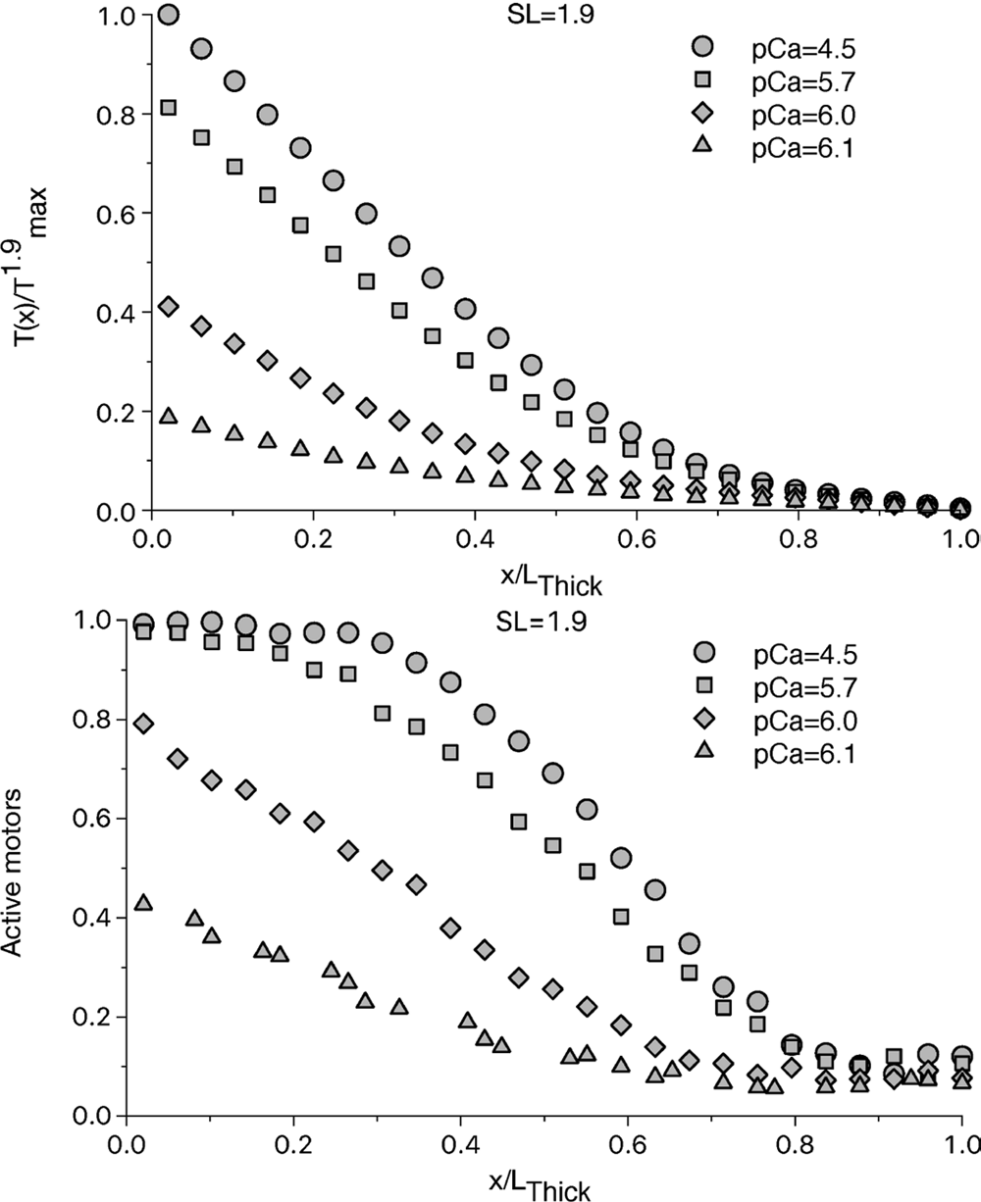
Relative tension and fraction of active motors along the thick filament at SL = 1.9 μm. The tension, normalized respect the maximum tension generated at the SL of 1.9 μm (T^1.9^_max_), at different [Ca^2+^] (upper panel) shows a non-linear increment along the thick filament, even at high [Ca^2+^], when the thin filament is fully activated. The non-linearity is due to a non-uniform activation of the motors along the thick filament (lower panel). At pCa = 4.5, there is a region of the thick filament, close to the M-line, which is fully activated, thanks to the high active tension generated by the motors upstream toward the Z-line. The low tension near the Z-line, instead, generates a region of slight more than constitutively activated motors, which survives even at this high level of [Ca^2+^]. The effect is more pronounced the less the thin filament is activated.

The simulated tension along the thick filament, averaged among 480 filaments, is shown in Fig. 1a for SL = 1.9 µm and at high [Ca^2+^], when the activation of the thin filament is complete. The simulated tension, normalized respect the maximum tension generated at the SL of 1.9 μm (T^1.9^_max_), has its maximum value at the beginning of the bare zone while it is almost zero toward the Z-line. This result is compatible with a uniform distribution of attached motors along the filament. However, the tension does not change linearly, having a higher steepness toward the M-line, and a much flatter shape toward the free end. This is an indication that the attached motors are not uniformly distributed, and this is due to the MS mechanism, which creates a synergic effect between the force exerted by the motors closer to the free end and the activation of the motors downstream, which in turn generates more force.

Our Monte-Carlo approach allows to evaluate, at each time step, the probability of each OFF myosin motors to switch ON, based on the actual tension acting on it. The simulation shows that, even at very high [Ca^2+^], when the total tension reaches its maximum and the thin filament is fully activated, there is a region of the thick filament where the local tension is not high enough to activate all the motors (Fig. 1b). Only the motors near the M-line are fully activated, while around the free end of the myosin backbone the population of active motors is not far from the constitutively ON level (we will call this region “constitutively activated”). With the chosen parameters, the thick filament activation has a sigmoidal distribution with an almost full activation up to one-third of L_Thick_, and a constitutive activation from 0.8 L_Thick_. In this region, the activation level is about 10%, higher than the constitutively ON level, because some motors are attached and generate a small force.

The situation is even more emphasized at intermediate level of [Ca^2+^], where the thin filament is not fully activated and a lower amount of active motors is recruited to generate force, sustaining the activation of motors downstream. Around the ECa_50_ (pCa_50_ ≈ 6) concentration, the constitutively activated region is extended to more than 0.7 L_Thick_ from the free end, and the thick filament is only partially activated everywhere. These results support the idea that the interaction between the two regulatory mechanisms is important in physiological contraction.

The current model ignores the presence of the myosin binding protein-C (MyBP-C). However, there are experimental indications that it has a stabilizing effect on the SRX state of myosin motors^23,24^. This effect and the peculiar localization of so-called “C-zone”, in the central region of the thick filament toward the M-line, may affect to some extent our results. Despite an extensive analysis of the MyBP-C influence is above the scope of the present work (see comments in the Discussion section), we can explore the effects of the induced non-uniform distribution of the constitutively active motors at the onset of the contraction. For simplicity, we define the C-zone as to be long about 450 nm starting from the M-line^25^ (the first half of the thick filament), and impose that the active state is completely suppressed below T_th1_. Then ON_min_ is increased in the half of the thick filament toward its free end to preserve the average number of constitutively active motors (3%, see Supplementary Fig. S1). Despite the tension dependent activation probability above T_th1_ is the same in the two regions and as before, the different initial distribution generates a wider fully activated region and a smaller constitutively activated region (Supplementary Fig. S2). The result is not trivial, because the tension dependent activation (above T_th1_) is much higher than ON_min_, and the steady state activation is reached after several cycles for each myosin head. We will discuss the implications later.

### Increasing passive tension improves the activation of the thick filament

**Figure 2:**
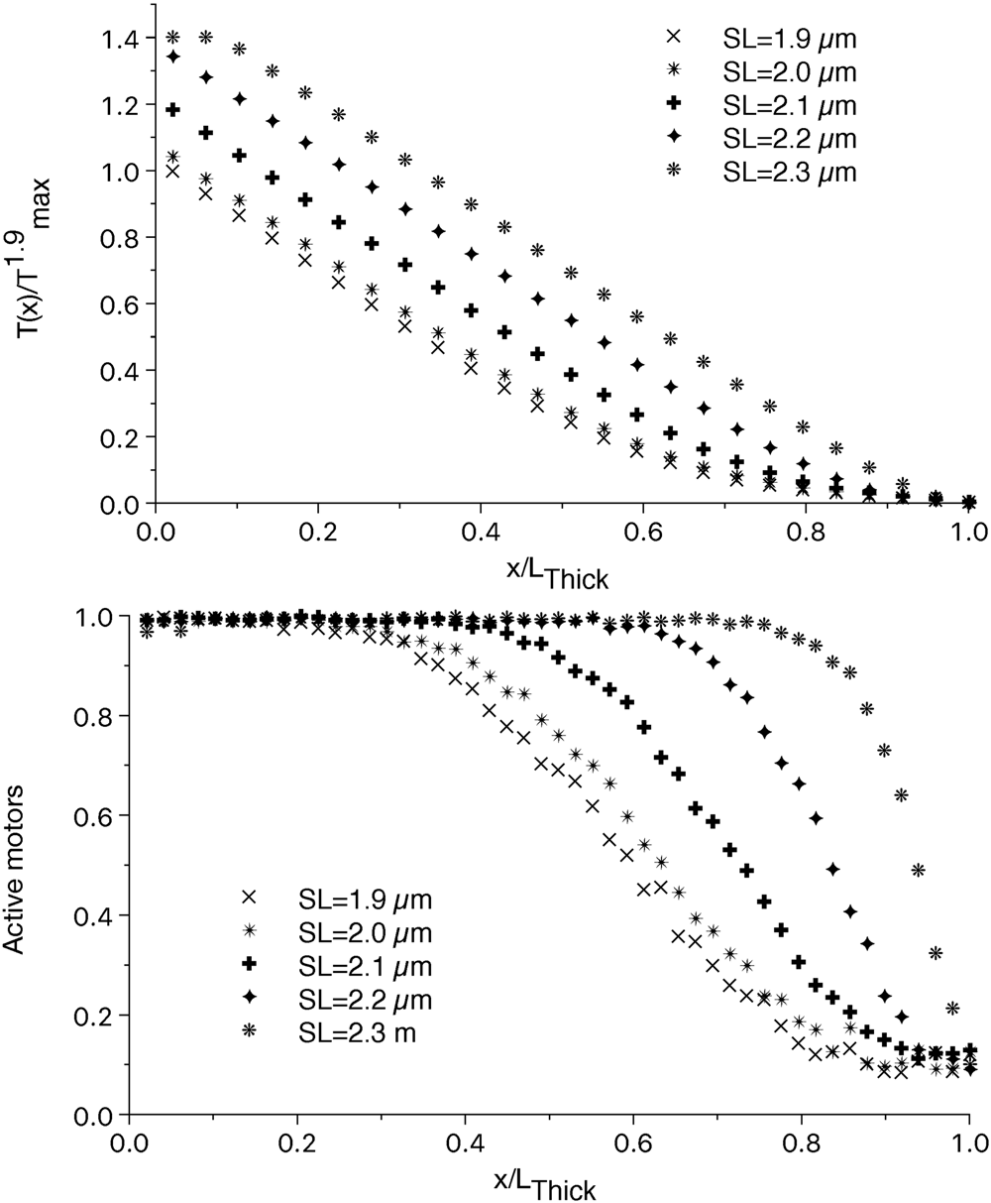
Relative tension and fraction of active motors along the thick filament at different SLs. The non-linear part of the tension along the thick filament is reduced at longer SLs (upper panel). This is due to the higher extension of fully activated region of the thick filament (lower panel) at longer SLs. At SL = 2.3 µm, the motors are almost fully activated, except for the very end of the thick filament. The small decrease in the activation close to the M-line at SL = 2.3 µm, is due to a partial loose of overlapping of the thin and thick filaments due the imposed geometry.

Active tension is always zero at the very end of the thick filament, and myosin motors cannot be activated by MS mechanism. On the contrary, passive tension acts uniformly along the filament. However, in relaxed cardiac muscle with no active tension, several experimental evidences indicate that the amount of constitutively ON motors does not change with the SL in the range between 1.9 and 2.3 µm. The estimation of the amount of active motors between these SLs does not show a statistical difference^7,26^, or it shows even a stabilization of the OFF state, probably due to the more compact configuration^15^. Following these indications, we imposed the first tension threshold T_th1_ equal to the passive tension generated by the titin at SL = 2.3 µm, so that in the relaxed muscle the amount of ON motors is the same at any SLs. However, when the thin filament allows for the attachment of motors, the higher tension generated by the titin sensibly increases the number of ON motors at longer SLs. At SL = 2.3 µm, the filament is almost fully activated up to 0.8 L_Thick_ and the constitutively activated region is almost negligible (see Fig. 2). Consequently, the number of attached motors and the force generated are higher, as shown in Fig. 2. The steepness of the sigmoidal curve between the almost fully activated and the constitutively activated regions, increases both with longer SLs and with higher [Ca^2+^].

When the different distribution of the constitutively active motors in the C-zone is considered, a similar effect as at shorter SLs is observed, despite the difference with the uniform distribution is smaller because of the already wide extension of the fully activated region (see Supplementary Fig. S2).

### The increase in T_max_ is quantitatively explained by the proposed mechanism and depends on the constitutively ON motors

**Figure 3:**
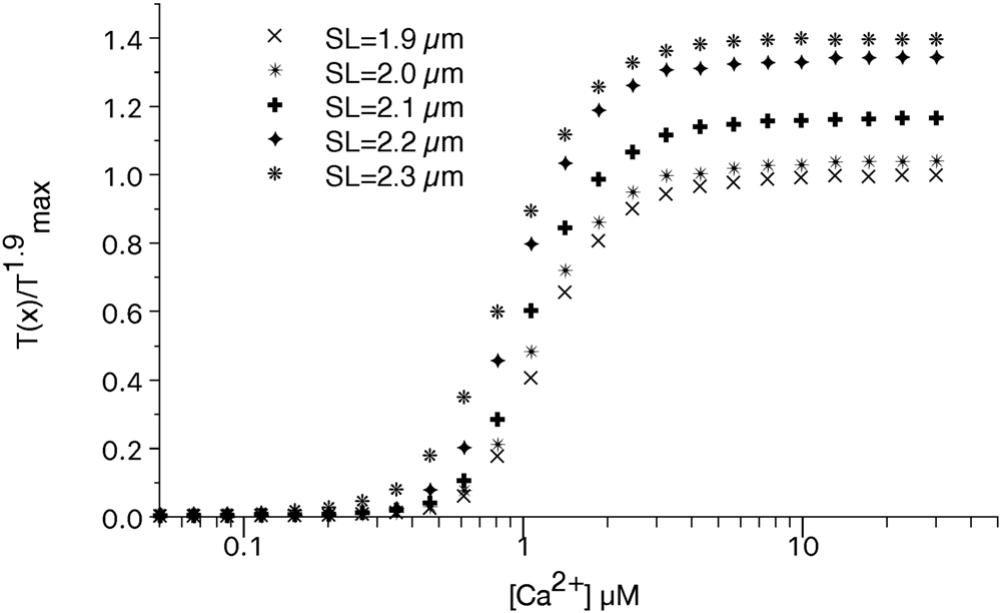
Normalized tension-[Ca^2+^] curves at different SLs. The simulated tensions at different [Ca^2+^] and SLs reproduce the experimental data showing that the proposed mechanism is able to explain the increment in T_max_ at longer SLs.

The experimental evidence of the LDA is usually given by the tension-[Ca^2+^] curves at different SLs. Our previous model was not able to reproduce the higher T_max_ because the “average-tension” approach activated all the motors along the filament when the total tension was over a given threshold. Instead, including the distribution of the tension along the thick filament, the free end of the myosin backbone is always populated by OFF myosin motors which can be activated by a higher passive tension. The active tension-[Ca^2+^] curves at different SLs, simulated by the model is reported in Fig. 3. The model numerically shows that the proposed mechanism is able to quantitatively explain the higher tension generated at longer SLs even when the thin filament is completely activated (high [Ca^2+^]). The parameters for the MS mechanism are defined to generate the experimentally observed increment in the total active tension which is about 40%^5,7^ for an increment of SL of 0.4 µm as in the simulation.

Despite at SL = 2.3 µm the passive tension is close to the first threshold, at low [Ca^2+^] the active tension is zero, because the thin filament is not enough activated. However, at longer SLs smaller amount of [Ca^2+^] are enough to generate some tension indicating a higher calcium sensitivity. The simulated Hill coefficient of the active tension-pCa curves, has a non-monotonical behavior with the increase of the SL, and its variation is within the 95% confidence bound for all the SLs except for SL = 2.3 µm where a non-optimal overlap of the filaments may influence this behavior (Table 1). Therefore, in our model, as in the experiments^5^, this parameter is not strongly related to the SL. The first component of the LDA, given by the shifting of the ECa_50_ toward lower values at longer SL, was reproduced in our previous model with the “average-tension” approach. It is also present in this model and the predicted shifting by the two models is the same, 0.15 pCa units, showing that the proposed mechanism doesn’t affect this component of the LDA. Notably, experimental data^7^ strongly suggest that this component of the LDA is based on the higher thin filament activation at longer SLs. However, we did not include such a dependence because the main goal of this work is to explore the second component of the LDA avoiding further complications of the model. Nevertheless, the predicted shift is close to that observed experimentally^5,7^ and the MS-mechanism seems to affect, at least to some extent, also this component of the LDA.

**Figure 4:**
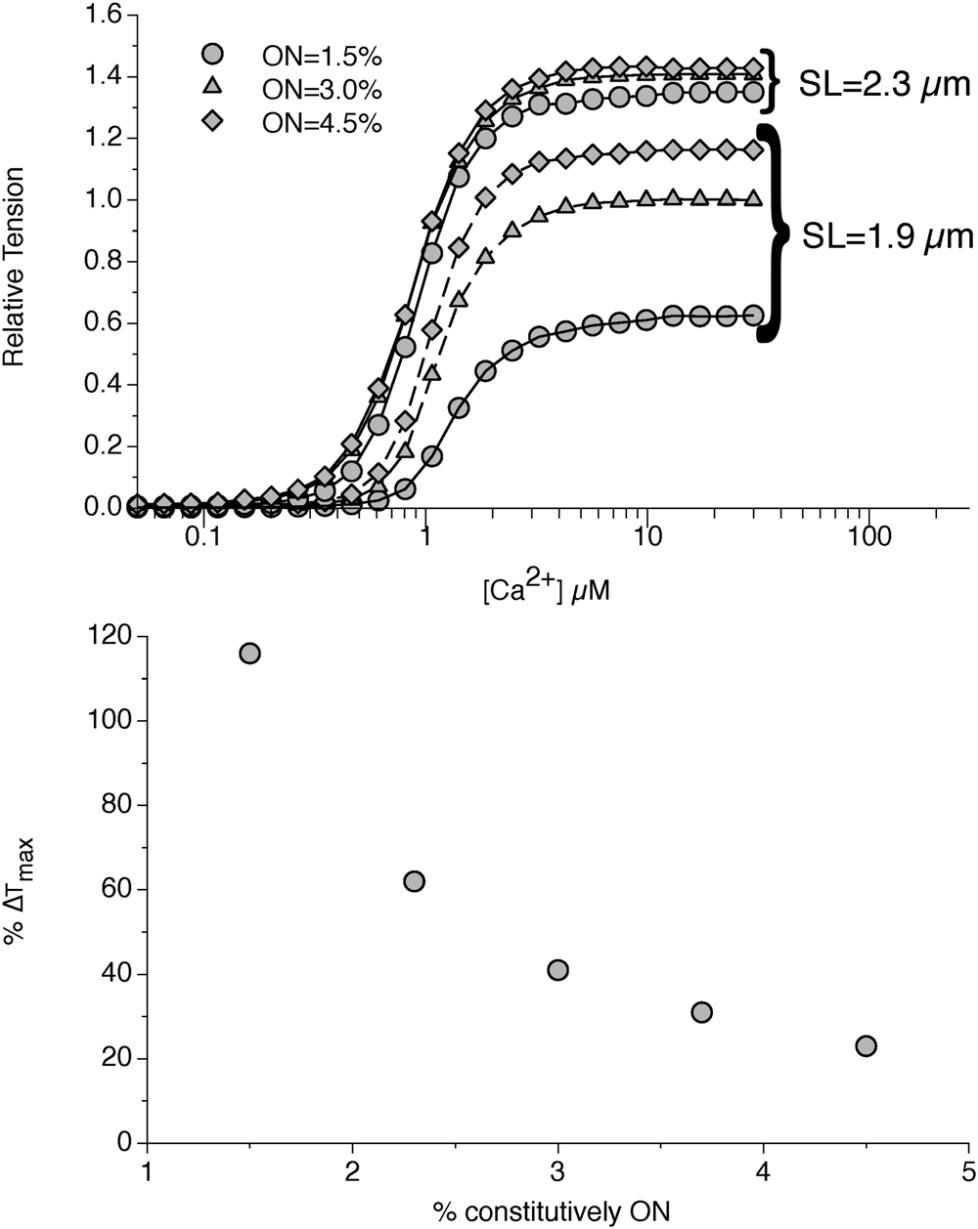
Influence of the constitutively ON motors on the increase in T_max_. At the given parameters for the MS mechanism, the increment in the SL, and consequently in the passive tension, affects more the increase in T_max_ at low SLs than at high SLs (1.9 and 2.3 µm in figure, upper panel). The effect is more pronounced at lower percentages of constitutively ON motors (lower panel), and tends to decrease at values higher than 5%. A value of 3% is predicted by the model to account for the 40% increment observed experimentally.

We can analyze the influence of the parameters of the MS mechanism on the T_max_-SL relationship. At high passive tensions (SL = 2.3 µm in the figure) the cooperative activation of the downstream motors is strong enough that, at high [Ca^2+^], almost all the motors are active even starting from a low amount of constitutively ON motors (Fig. 4a for SLs 1.9 and 2.3 µm). However, at lower SLs (SL = 1.9 µm in the figure) the cooperation is much more difficult. In this situation, the number of constitutively ON motors strongly influences the value of T_max_. In Fig. 4b is shown the percentage change of ΔT_max_ = T^1.9^_max_ − T^2.3^_max_, the maximum tensions generated at high [Ca^2+^] at SL of 1.9 μm and 2.3 μm respectively, as a function of the percentage of constitutively ON motors. Very small differences in the ON_min_ value generates an important variation in ΔT_max_ and about 3% of constitutively ON motors accounts for the required increase in T_max_. Notably, this value is close to the value of 5% hypothesized for skeletal muscle by Linari and colleagues^14^. However, the model predicts a strong dependence of ΔT_max_ on the amount of the constitutively ON motors up to 4–5% and a marked decrease around this value. This finding may be important to define an experimental protocol to test our hypothesis and numerical results, but it is worth to note that a very low amount (1–4) of attached motors per thick filament has been estimated in unloaded shortening^27^. ΔT_max_ is also decreased by the hypothesized effect of the MyBP-C because of the above mentioned reduction of the *reservoir* in such a situation (see Supplementary Fig. S3).

### The influence of the proposed mechanism on the Frank Starling relationship may be limited in physiological contraction

We want to estimate the influence that the higher T_max_ at longer SLs has on the Frank-Starling mechanism *in-vivo*, so we included the single fiber model, described before, into a three dimensional, rotationally symmetric, FE model of the rat left ventricle. Using this approach in our previous work, we have already shown that the first component of the LDA, i.e. the higher calcium sensitivity at low-medium [Ca^2+^], explains at least partially the increase in the ejection volume (EV) at higher preloads^20^. However, that increase did not quantitatively match the experimentally observed values. One possible explanation is that the previous model failed to reproduce the second component of the LDA, being T_max_ independent from SL. We can now explore the influence of this component of the LDA, comparing the EV-preload curves predicted form the current and the previous model.

**Figure 5:**
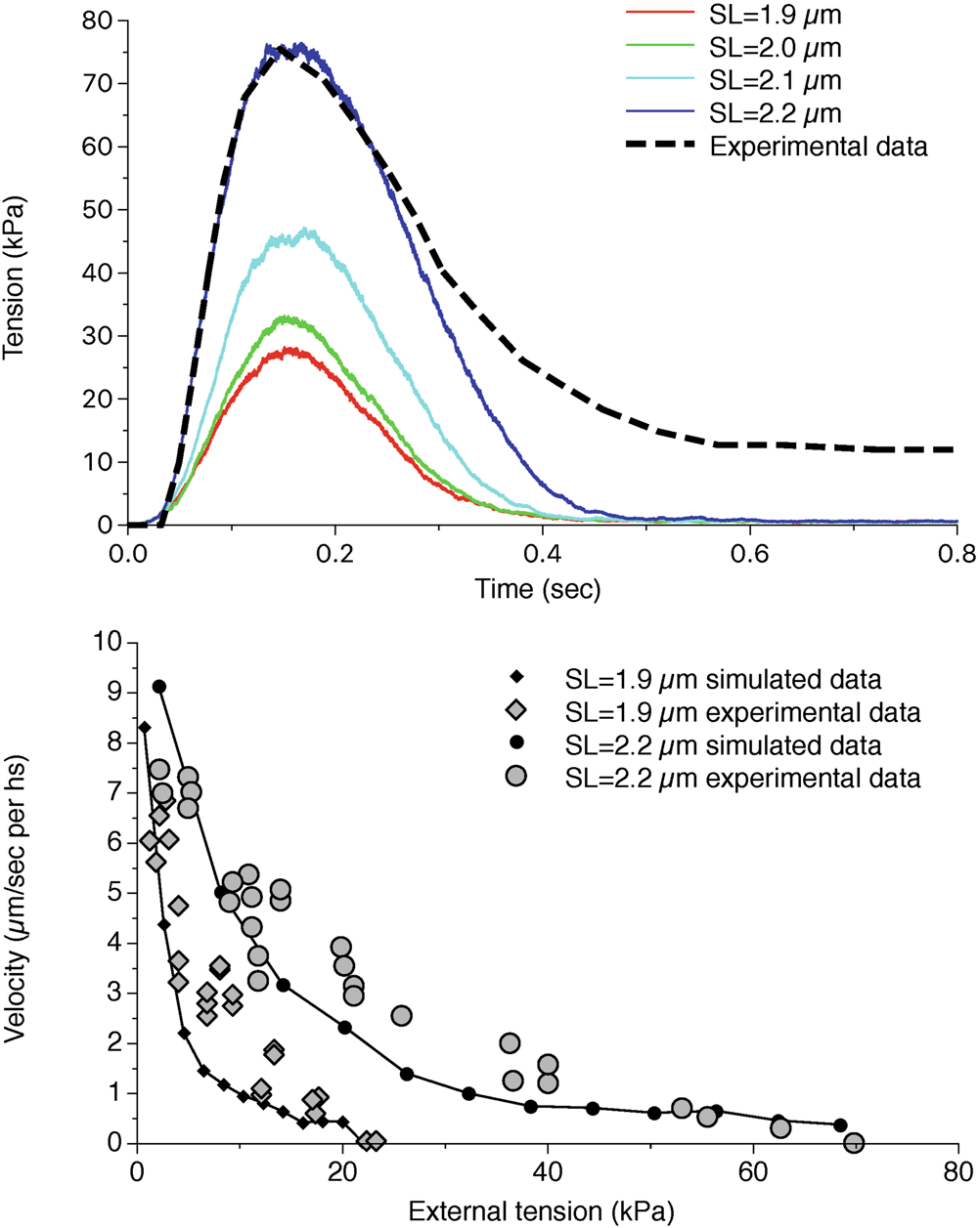
Tension during a twitch and tension-velocity curves for the single trabecula model at different SLs. Attachment-detachment rates and power-stroke energetic landscape for myosin motors are fitted to experimental data for the tension-time curves during a twitch (upper panel) and for the isotonic velocity of contraction (lower panel). The different behaviours at different SLs are matched. Dashed line in the upper figure is obtained by extrapolation of experimental data from Caremani *et al*.^4^ and adapted to the experimental maximum tension at SL = 2.2 µm.

The parameters for the working cycle are defined to match the experimental behavior of intact rat trabecula^4^. Similarly to previous model, the titin-mediated thick filament activation is able to produce a higher peak tension during a twitch for longer SLs (Fig. 5a), generating a more than two-fold increase in the peak tension at 2.2 µm compared to 1.9 µm, as observed experimentally, while the passive tension increases by only 10% of the highest generated tension. Moreover, the simulation of the isotonic contraction (Fig. 5b) shows that the maximum velocity is independent on SLs, as well as the curvature of the tension-velocity curve, because the mechanokinetics of the force generating motors is the same, and the MS mechanism acts mainly as a regulator of their number^4,20^.

**Figure 6:**
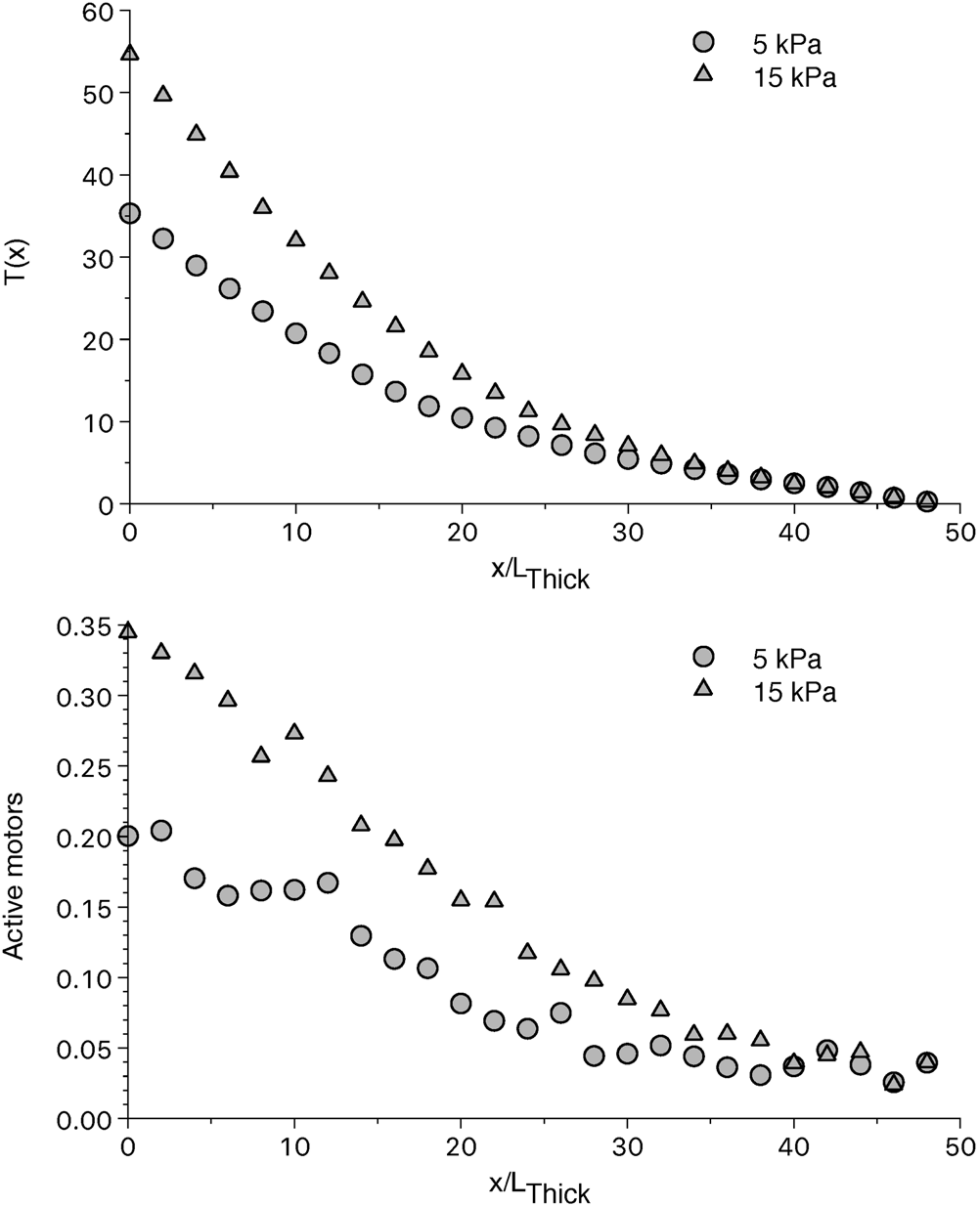
Tension and fraction of active motors along the thick filament averaged among all the left ventricle elements at different preloads. The average tension (upper panel) increases from the thick filament free end toward the M-line in a non-linear manner. As in the half-sarcomere model, this is due to the non-uniform activation of the myosin motors (lower panel). Increasing the preload, increases the active motors and consequently the tension generated. Data is taken 96 ms after the beginning of the systolic phase.

**Figure 7:**
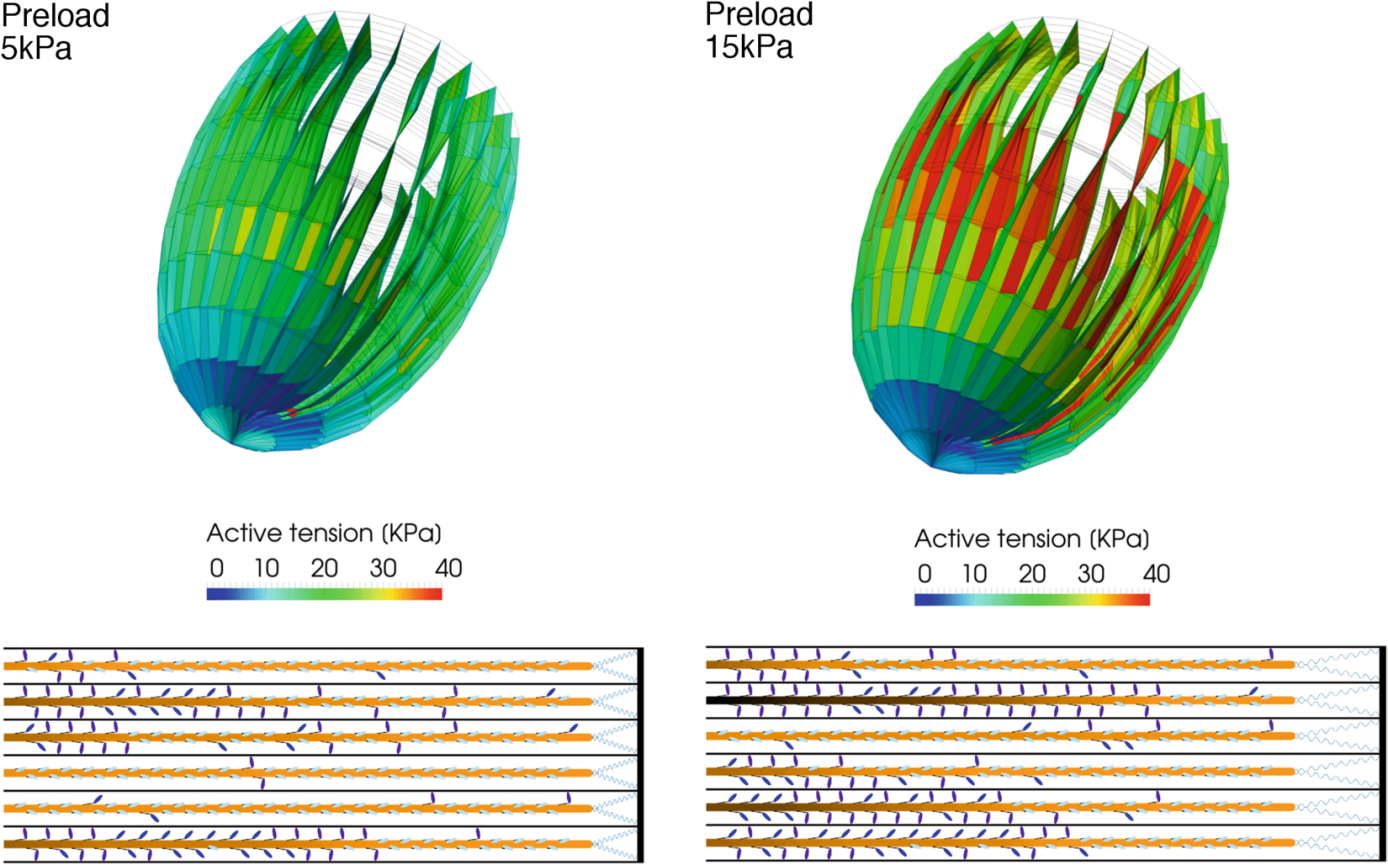
Ventricle model and a portion of a representative sarcomere at two different preloads. The FE model of the left ventricle (upper part of the figure), shows a higher tension in the elements when preload increases from 5 to 15 kPa (left and right part of the figure respectively), especially in the central region of the ventricle wall. The higher tension is due to a longer SL, which activate a higher number of myosin motors through the higher passive tension generated by the titin (lower part of the figure). Myosin motors in figure are shown in their simulated state during a beat: OFF detached state (clear blue), ON detached state (blue), and attached (violet). Data is taken 96 ms after the beginning of the systolic phase.

We introduce this fiber model into the left ventricle FE model and estimate the influence of the proposed mechanism on the macroscopic ventricle behavior, imposing increasing preloads, and consequently end diastolic volumes, at the onset of the contraction. In average, a higher diastolic volume generates a higher tension in the ventricle sarcomeres, which is due to a higher number of active motors at the beginning of the systolic phase (Fig. 6). As in the half-sarcomere model, these averaged values are non-uniformly distributed along the thick filament because of the proposed mechanism. The left ventricle model shows a higher tension especially in the mid-portion of the ventricle (Fig. 7). The higher tension at higher preloads was present also in our previous model but now the distribution of the active myosin is decreasing along the filaments, and a more or less extended region characterized by an almost constitutive activation can be seen toward the Z-line as shown by a representative half-sarcomere model shown in figure. It is interesting to note that the model predicts that some filaments may be always deactivated, leaving other filaments to fully support the required tension (Fig. 7). This phenomenon may be present in the physiological contraction, even though it is probably not so clean-cut because of the compliance and interaction of the filaments, not included in this model.

**Figure 8:**
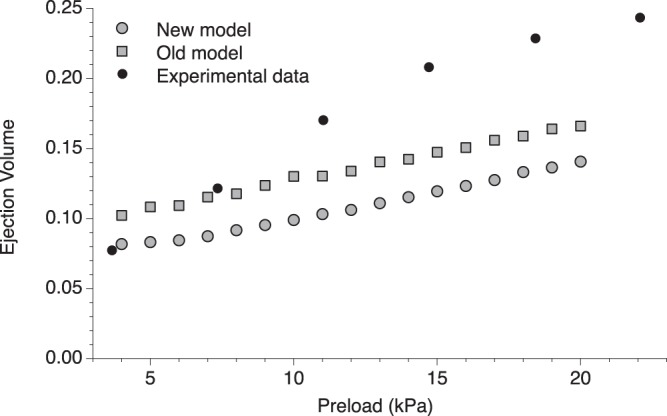
Simulated ejection volume at different preloads (grey circles) and comparison with experimental data^40^ for rat myocardium (filled dots) and old model (grey boxes). The new model is able to predict an increase in the ejection volume at higher preloads, due to the higher tension generated at longer SLs. However, the increase is not as much as observed experimentally, indicating that other mechanisms are involved in the Frank-Starling law of the heart. More interestingly, the increase in the EV is very similar to the one predicted from our previous model which generates the same T_max_ for all SLs (the two simulated data are not superimposed for the intrinsic differences between the models, in the new model the ejection volume at 5 kPa has been chosen for the initial fitting of the FE parameters). This result opens the question about the relative importance of the second component of the LDA in physiological situations.

The extension of this constitutively activated region may be important to increase the EV at higher preloads. This aspect is not trivial, since the activation during heart contraction is only partial, with sub-maximal [Ca^2+^], and the second component of the LDA may be not so important during physiological contraction. The EVs simulated at different preloads are reported in Fig. 8. Through the increased recruitment of active motors due to the MS mechanism more blood is ejected when the preload increases. However, when compared to the experimental data, the model fails to fully explain the Frank-Starling relationship, showing a smaller slope in the curve. Moreover, comparing the simulation to that obtained by the model without the second component of the LDA, we can see the striking similarity between the slopes of the two curves. Within the limits of the model, we can quantitatively estimate the relative influence of the two components of the LDA on the Frank-Starling relationship, comparing the increment of the ejection volume between 5 kPa and 20 kPa preloads ΔEV in the two models and the experimental data. The higher calcium sensitivity at intermediate [Ca^2+^] shown by longer SLs, the sole LDA component present in the old model, cannot fully explain the experimental increment (ΔEV_exp_ = 0.138 ml) but it can still account for about one-half of it (ΔEV_old_ = 0.058 ml). The increment predicted by the new model, which quantitatively reproduces both components of the LDA including the higher Tmax at longer SLs and at saturating [Ca^2+^], is almost coincident to the old model (ΔEV_new_ = 0.057 ml). In fact, the analysis of the tension generated by each fiber, shows that the region of interest of the higher T_max_ is never reached during the simulated contraction, and the second component of LDA is only marginally, if at all, affecting the Frank Starling relation. Our simulations indicate that in physiological conditions the cardiac muscle cells work in the low-medium [Ca^2+^] region of the force-[Ca^2+^] relationship. Therefore, at higher preloads, while the shifting of ECa_50_ at longer SLs observed at the single fiber level exerts a role in the whole organ behavior, the higher T_max_ has a limited or absent effect.

This relatively small effect of the second component of the LDA on the EV, may be due to the particular choice of the parameters, because they are based on experimental data both on skinned (tension-[Ca^2+^]) or intact (twitch and isometric contraction) rat trabeculae and not on *in-vivo* data. The behavior in intact heart may be different, for instance, because of material damage in extracting the specimen or different lattice spacing. It is known, for example, that the tension-[Ca^2+^] curve in intact fibers is shifted toward lower [Ca^2+^] and with a higher Hill coefficient^28^, which may lead to an influence of the higher T_max_ at longer SLs. We explored this situation with a simple modification of the parameters for the RU activation, increasing its sensitivity to calcium ions. However, this sensitivity also reduced the diastolic refilling because of residual tensions and the model was not able to increase the EV-preload slope with this modification (not shown). Another aspect which may improve the EV at higher preloads is the amount of constitutively ON motors, since we have shown that in the single fiber it has a major role in defining ΔT_max_. We modified the ON_min_ value as done before for the fiber simulations. At each value correspond a different EV because it also influences the tension generated, however, we report the effect on the slope of the EV-preload curve in Table 2. Decreasing the number of constitutively ON motors increases slightly the slope of the curve, supporting the conclusions obtained for the single fiber. However, even at very low values of ON_min_ the predicted ΔEV is smaller than the one extrapolated by the experimental data (ΔEV = 0.138 ml), further supporting the idea that the second component of LDA is weakly influencing the Frank-Starling relation.

**Table 2:** Ejection volumes and their difference at 5 and 20 kPa preloads for different values of constitutively ON motors.

ON_min_	EV at 5 kPa (ml)	EV at 20 kPa (ml)	ΔEV (ml)
1.5%	0.041	0.106	0.065
2.3%	0.062	0.125	0.063
3.0%	0.083	0.141	0.058
3.7%	0.105	0.155	0.050
4.5%	0.124	0.167	0.044

## Discussion

We presented a mathematical model of cardiac muscle fiber, which includes the recently discovered dual filaments regulatory systems, and is based on the hypothesis that the local tension is the only cause of the myosin motors activation. Under this hypothesis, the results show that the thick filament is not fully activated even at high [Ca^2+^], and that it can be separated into three regions: a fully activated region, close to the M-line, a constitutively activated region, close to the Z-line, and an intermediate region of activation. The activation has a sigmoidal shape which shows the cooperativity between the tension generated by the upstream motors, and activation of the downstream motors. Higher passive tensions exerted by titin reduce the extension of the constitutively activated region and increase the extension of the fully activated one, increasing T_max_. Notably, a possible consequence of the non-uniform activation of the thick filament predicted by the model, is the steeper-than-expected ascending limb in the force-length relationship observed experimentally^6^, since the double overlapping of thin filaments at very short SLs would first destroy the attachment in the region of full activation. Under our assumption, the model predicts that the amount of constitutively ON motors is a key determinant of the increase in T_max_ at longer SLs, and that the observed values are compatible with a 3% of active motors in relaxed muscle. Perturbing this value and analyzing the effect on the T_max_ at different SLs, would provide an experimental test for our hypothesis. We also applied our model to a left ventricle finite element model, showing that while the proposed mechanism is able to quantitatively explain the higher T_max_ in the tension-pCa curve, this feature may be not crucial for the Frank-Starling relationship between the EV and the preload in physiological situations. However, linking the skinned fibers data to the *in-vivo* behavior is a challenging task for the experimentalists, and the numerical simulations, whilst can be a powerful tool to bridge the two situations, suffer similar limits that must be highlighted. The steepness of the tension-pCa curve, indicated by n_H_, is close to the one observed in skinned trabecula, but it is lower than the one observed in intact cells. Even though in the model, as in the experiments, n_H_ is not affected by the SL, a higher cooperativity can increase the influence of the higher T_max_ in physiological contractions. As another limit, in this work we focused on the effect of the MS mechanism on LDA and no SL effect has been included for the thin filament activation. This effect exists, as shown experimentally^7^. However, the thin filament is fully activated at high [Ca^2+^] and this dependence would not affect our conclusion on T_max_. Regarding the ascending limb of the force-length curve, other mechanisms, not considered here, play a role in this slope^29^, and a detailed analysis of this relationship is above the scope of this work.

Finally, the absence of other proteins in this simplified model, and in particular of the myosin binding protein C (MyBP-C), requires an extended discussion. Muscle contraction is based on the concerted action of several proteins, and despite their effects are often not completely understood, their absence is likely to explain the lower than observed relation between EV and preload simulated by the model. On the other side, this aspect also highlights the potential impact of the predicted non-uniform activation induced by the MS mechanism on muscle regulation, considering the MyBP-C peculiar position, close to the M-line, where the thick filament results more activated. Moreover, MyBP-C tends to suppress the activation of the SRX motors, but also to increase the activation of the thin filament^23,24^. Therefore, in the C-zone the activation level of the thick filament should be decreased by the higher stability of the SRX state but increased by the higher tension that fewer active motors can generate with a more activated thin filament. These effects would also be affected by the calcium diffusion inside the cell during each beat, and the position of the ryanodine receptors and sarcoplasmic reticulum Ca^2+^-ATPase pumps can have an active role on the non-uniform activation of the filaments^25,30^. A detailed analysis of these aspects is beside the scope of the present work and are left for future research. However, we analyzed the potential role that the MyBP-C can have in the distribution of the constitutively active heads, the “sentinels” of the relaxed thick filament which must transmit the information about the thin filament activation to other SRX heads. We have shown that, kept the number of sentinels and other parameters constant, when they are concentrated toward the free-end of the thick filament, its activation is higher than when they are randomly and uniformly distributed. In particular, the simulations show that its effect in the relaxed muscle is influencing the whole contraction, even in isometric conditions for several hundred of milliseconds, despite the tension mediated activation is much stronger and the motors undergo to several ATP-cycles. These results show that the MS mechanism has an important role in the cooperative transition imposed by the MyBP-C along the thick filament and supports the idea that it may act to regulate the contraction more than to uniformly distribute it^23^.

Despite the model limits, the results presented in this work support the idea that the higher T_max_ exerted at longer SLs when the thin filament is fully activated, is associated with a titin-mediated activation of the thick filament through the MS mechanism.

**Figure 9:**
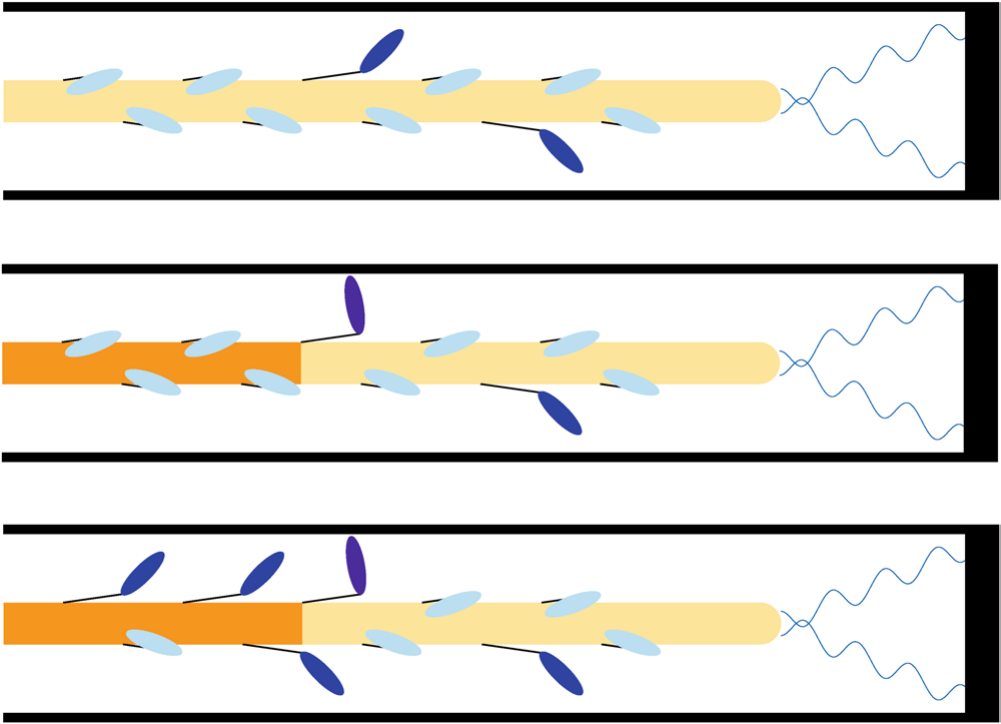
Proposed mechanism. Myosin motors are in a detached OFF state (clear blue, folded on the thick filament), a ON detached state (blue), or an attached state which generates force (violet) At the onset of the contraction the motors have the same probability to be active (upper panel). However, when an active motor strongly attaches to the thin filament, it generates tension only on the portion of the thick filament toward the M-line (intermediate panel). The higher tension generates a higher probability of the downstream motors to be activated (lower panel) and then to produce force, generating the reservoir toward the Z-line.

Our previous model based on a uniform distribution of the tension along the thick filament failed to match the higher T_max_ at higher SLs because, at least with that set of parameters, the thick filament was fully activated at high [Ca^2+^] even at low SLs. The higher T_max_ at longer SLs can be generated by the thick filament activation if it is only partially activated at low SLs. However, the increase in T_max_ for SL from 1.9 µm to 2.3 µm is about 40%^7^, and this may be hardly explained by the passive tension considering its relative low value respect to the active tension at high [Ca^2+^]. We propose an explanation based on the different distribution of active and passive tension associated with the sarcomere ultrastructure. The smallest contractile unit in muscle is the half-sarcomere, because the symmetric organization of the motors leads to the shifting of both Z-lines toward the central M-line in the whole sarcomere. Consequently, the active tension along the thick filament, increases starting from the thick filament free-end toward the sarcomere center, being the sum of all the attached motors. We have shown that, even in the hypothesis that at the onset of the contraction the probability for a myosin to switch ON and to generate force is the same (ON_min_) along the thick filament (Fig. 9), once a motor rises toward the thin filament and produces force, it increases the probability to activate all the motors downstream toward the M-line but not those upstream, leaving a region of constitutively activated motors near the free end of the thick filament. During the contraction the model predicts the formation of a *reservoir* of OFF motors closer to the Z-line predicting a full activation only toward the M-line. The *reservoir* is a consequence of our hypothesis on the MS mechanism, which, in this model, activates the motors proportionally to the local tension. The *reservoir* can then be activated, when needed, through an increase in the tension in that region. We hypothesized that titin protein generates tension before interlacing with the myosin backbone. Therefore, differently from active tension, passive tension is uniformly distributed along the thick filament, even in its free end, and acts directly on the *reservoir* of myosin motors.

A relationship between the tension and the activation of myosin motors has been shown experimentally for both active and passive tension in cardiac and skeletal muscles^4,7,14,18^. However, it is unclear if active tension is the cause or the consequence of the switching ON of the motors. The first case, hypothesized in this model, takes place if the tension acting on a myosin might loosen the stability of the interacting-heads motif, destabilizing the super-relaxed state, and generating, in turn, a higher tension. Instead, a different situation would be generated if, for instance, the uniformity of the interacting-heads motif is a crucial factor for keeping the whole thick filament deactivated. Here, when the thin filament is activated, a constitutively ON motor attaches to it, both generating force and destabilizing the SRX in the nearest neighbor proteins because of the sole interruption of the interacting-heads motif. In this case, the activation of the thick filament would be uniformly distributed, and no *reservoir* would be possible. Although different mechanisms could prevent the complete activation of the thick filament leading to the increase of T_max_ at longer SLs even in this case, other evidences suggest that the tension directly cause the activation^18^, and passive tension effect cannot be explained by the nearest neighbor influence. Moreover, during unloaded contraction a quite rapid recovery of the SRX has been shown^14^. Finally, the mechanism proposed in this work is rather simple, and the simulated behavior, which matches the experimental one, suggests that the tension has an active role.

Frank-Starling law is a crucial property of physiological heart contraction and an unpaired relationship between the EV and the preload is associated with cardiomyopathy and heart failure^3,31^. It has been intensively studied for more than a century as well as its cellular counterpart, the LDA, however its molecular mechanisms remain largely unknown. In the last few years, the discoveries of MS-mechanism and dual filaments activation are promising to improve our knowledge on it and to open the ways to new clinical treatment of cardiomyopathies. In this work we have proposed a relatively simple explanation of the mechanism which relates the thick filament activation to the second component of the LDA through the MS-mechanism, making a step toward the full comprehension of the molecular basis of the Frank-Starling law of the heart. Moreover, recent evidences link the SRX to hypertrophic cardiomyopathies^32,33^ and the proposed mechanism, if experimentally proved, may help in the definition of their clinical treatment.

## Material and Methods

### Fiber model

The half-sarcomere model presented in this paper is an evolution of the one proposed in our previous work^20^ and we reference to it for a detailed description, reporting here the major features and the novelties. 480 thin and thick filaments are superimposed, being the latter fixed in the reference system and the former attached to a common Z-line. Filaments can slide past each others under the force exerted by myosin heads spaced every 14.3 nm on the myosin backbone. Thin filaments are 1 µm long and thick filaments are 1.6 µm long with a bare zone of 0.2 µm^34,35^, then overlapping is optimal between SLs of 1.9 µm and 2.2 µm.

Rate constants in the resting cycle regulate the activation of the thick filament, with a constant switching OFF rate, k_ON-OFF_, and a tension dependent switching ON rate, k_OFF-ON_(T), already described in the text. The major difference with previous model is in the definition of the tension dependence of k_OFF-ON_ which here is based on the actual tension acting on each myosin motor at each time step. Then, the state of each myosin motor influences the transition rates of all the motors which are downstream in the thick filament. To ensure this analysis we used a Monte-Carlo approach, defining a random sampling to simulate the behavior of each motor at each time step. This method allows to overcome the limits of the ordinary differential equations approach, used in other models, which approximate the actual conditions through their average behavior along the filaments. The Monte-Carlo method is also used in the working cycle to simulate the probabilities of attachment, detachment and performing the power stroke which depend on the tension acting on each motor^21,36–38^, as well as to include the effect of thermal fluctuations as previously described^22^.

Thin filaments are activated by the [Ca^2+^] through the opening of the Troponin-Tropomyosin regulatory units (RU) placed every 36 nm, which can be inactive, partially active or active. We based the thin filament activation on the experimental data obtained by Zhang and co-workers^7^. Then the RU intermediate state has been considered negligible to increase the basic cooperativity up to its theoretical maximum value (n^b^_H_ = 2 for the thin filament). To match the thin filament activation when the attached myosin are inhibited by the presence of blebbistatin (n^d^_H_ = 2.74 at SL = 1.9 µm) we also included a RU-RU cooperativity^39^. In the single trabecula simulations, we did not include other sources of cooperativity for the thin filament activation, to focus on the effect of the thick filament activation. To maximize the MS mechanism on the Frank-Starling relationship in the ventricle model, instead, we considered also the increase in the thin filament cooperativity in the presence of active force (n^a^_H_ = 3.29 in the experiments^7^) through a XB-RU cooperativity, where an attached myosin motor increases the probability of the RU to be activated by a calcium ion. As mentioned in the text, we did not include the SL effect on the thin filament activation to focus on the thick filament activation effect.

Tension-[Ca^2+^] curves and thin filament activation-[Ca^2+^] curves are fitted through a Hill equation (see equation 1) with three parameters: T_max_, the maximum tension generated at high [Ca^2+^] (or the maximum activation for the thin filament), ECa_50_, the [Ca^2+^] required to generate one-half of T_max_, and n_H_, the Hill coefficient. The fitting was made by Matlab® Fitting toolbox, with a non-linear least-squares method.

### Ventricle model

Ventricle model is the same as in our previous work^20^. The implementation of the fiber model in the FE ventricle model is extensively described in the Supplementary Methods and Supplementary Fig. S4. Importantly, the tension-dependent release of resting motors in the FE model is exactly the same as in the fiber model.

## Electronic supplementary material

Supplementary informations
