# Supplementary material for: Proposed mechanism for the length dependence of the force developed in maximally activated muscles
Source: Sci Rep. 2019 Feb 4;9:1317. doi: 10.1038/s41598-018-36706-4 (PMC6362285; doi:10.1038/s41598-018-36706-4)
Supplement: Supplementary file 1 — Supplementary informations [file 41598_2018_36706_MOESM1_ESM.pdf]

Proposed mechanism for the length dependence of the force developed in maximally activated muscles

Lorenzo Marcucci, Takumi Washio, Toshio Yanagida

Supplementary Methods

Ventricular model

The beating heart simulation is performed with a rotationally symmetric left ventricle model, as shown in Fig. S4. The ventricular wall is simultaneously stimulated by the same time profile of calcium ion concentration as the single twitch simulation. The momentum equation of the heart wall is given as follows.

$$\int_{\Omega_0} \boldsymbol{\delta\dot{u}} \cdot \rho \boldsymbol{\ddot{u}} \, d\Omega + \int_{\Omega_0} \frac{\partial \boldsymbol{\delta\dot{u}}}{\partial \boldsymbol{X}} : \boldsymbol{\Pi}^T \, d\Omega = P_{\mathrm{Cav}} \int_{\Gamma_t} \boldsymbol{\delta\dot{u}} \cdot \boldsymbol{n} \, d\Gamma$$

Here, Ω_0 is the ventricle wall in the unloaded condition, $\boldsymbol{u} = \boldsymbol{u}(\boldsymbol{X}, t)$ is the displacement of the material point $\boldsymbol{X} \in \Omega_0$ at time t, and ρ is the density of the heart muscle ($\rho = 1.366 \mathrm{Kg/m^3}$). Γ_t is the blood–wall interface at time t and $\boldsymbol{n}$ is the outward normal vector on Γ_t. P_{Cav} is the intracavity pressure, which is determined by combining the conservation equation of the blood volume:

$$\dot{V}_{\mathrm{Cav}} = F_{in} - F_{out}$$

where V_{Cav} is the intracavity volume, F_{in} is the inflow from the preload and the F_{out} is the outflow to the afterload. These flows are determined from the relationship between the intracavity pressure P_{Cav} and the pressure behind the valves, represented by the rectifiers in Fig. S4.

$\boldsymbol{\Pi}$ is the first Piola-Kirchhoff stress tensor, composed of active and passive stresses:

$$\boldsymbol{\Pi} = \boldsymbol{\Pi}_{\mathrm{act}} + \boldsymbol{\Pi}_{\mathrm{pas}}$$

The active stress $\boldsymbol{\Pi}_{\mathrm{act}}$ is derived from the active tension T_{act} with

$$\boldsymbol{\Pi}_{\mathrm{act}} = \frac{T_{act}}{\lambda} \boldsymbol{f} \otimes \boldsymbol{f} \cdot \boldsymbol{F}^T$$

where $\boldsymbol{F} = \boldsymbol{I} + \partial \boldsymbol{u} / \partial \boldsymbol{X}$ is the deformation gradient tensor and $\lambda = \|\boldsymbol{F}\boldsymbol{f}\|$ is the stretch along the myofibril fiber direction[1]. The fiber orientation is twisted from 90 to -60 degrees in the circumferential direction, from the internal wall to the outer wall, along the transmural direction. The active tension T_{act} per unit area in the unloaded configuration, the nominal stress, is given by

$$T_{act} = \frac{R_S}{SA_0} \frac{1}{n_F} \sum_{j=1}^{n_F} \sum_{i=1}^{n_M} \delta_{A,i,j} F_M(\xi_{i,j})$$

Here, the dynamics Monte Carlo simulation for a half-sarcomere model composed of n_F=12 pairs of thick and thin filaments are performed in each finite element to give the individual spring force

$$F_M(\xi_{i,j}) = 1/2k\, {\xi_{i,j}}^2$$

with strain $\xi_{i,j}$ of the attached myosins ($\delta_{A,i,j} = 1$). The stiffness is supposed asymmetric with values of 2 pN/nm in tension and 0.4 pN/nm in compression (for details see Marcucci et al.[2]). The spring strain $\xi_{i,j}$ at time t was given by considering the power stroke distance $ps_{i,j}(t)$ and the sliding distance from the most recent attachment time $t_{A,i,j}$

$$\xi_{i,j}(t) = \xi_{i,j}(t_{A,i,j}) + ps_{i,j}(t) + \frac{1}{2} SL_0 \left(\lambda(t) - \lambda(t_{A,i,j})\right), \qquad \mathrm{t} > t_{A,i,j}$$

Here, SL_0 is the sarcomere length in the unloaded condition, and the initial strain $\xi_{i,j}(t_{A,i,j})$ at the attachment was randomly chosen from the Boltzmann distribution determined by the strain energy of the spring. The above dependence of the spring strain on the stretch λ of the finite element is the feedback from the finite element ventricle model to the Monte Carlo molecular model. On each thick filament, there are n_M myosin molecules, which underwent repeated attachment and detachment with the thin filament. The value of n_M = 49 takes into account the geometry of the sarcomere and the bi-dimensional simplification used in the model as described in our previous work[2]. SA_0 is the cross-sectional area of a single thin filament and R_S=0.5

denotes the volume density of the muscle fibers within trabecula. The details of how to deal numerically this coupling in the simulation is given in our previous work[1].
The passive stress is determined by the potential:

$$W_{\mathrm{pas}} = W_{\mathrm{Mooney}} + \kappa(\det(\boldsymbol{F}) - 1)^2 + W_{\mathrm{titin}}(\lambda) + W_{\mathrm{collagen}}(\lambda)$$

The first term represents the homogeneous potential of the Mooney-Rivlin body:

$$W_{\mathrm{Mooney}} = c_1(\tilde{I}_1 - 3) + c_2(\tilde{I}_1 - 3)^2 + c_3(\tilde{I}_1 - 3)^2(\tilde{I}_2 - 3)$$

Here, $\tilde{I}_1 = \det(\boldsymbol{C})^{-1/3} Tr(\boldsymbol{C})$, $\tilde{I}_2 = \det(\boldsymbol{C})^{-2/3}\left(Tr(\boldsymbol{C})^2 - Tr(\boldsymbol{C}^2)\right)$ represents reduced invariant determined by the right Cauchy-Green deformation tensor $\boldsymbol{C} = \boldsymbol{F}^T\boldsymbol{F}$ (c_1=40Pa, c_2=2000Pa, c_3=40Pa). The second term is the potential for volumetric deformation ($\kappa = 10^6\mathrm{Pa}$). The third and fourth terms represent the potentials given by titin and collagen. These potentials are functions of the stretch λ along the fiber direction.

Reference:

1. Washio, T. *et al.* Ventricular fiber optimization utilizing the branching structure: Ventricular fiber optimization utilizing the branching structure. *Int. J. Numer. Methods Biomed. Eng.* **32,** e02753 (2016).

2. Marcucci, L., Washio, T. & Yanagida, T. Titin-mediated thick filament activation, through a mechanosensing mechanism, introduces sarcomere-length dependencies in mathematical models of rat trabecula and whole ventricle. *Sci. Rep.* **7,** 5546 (2017).

Supplementary Figures:

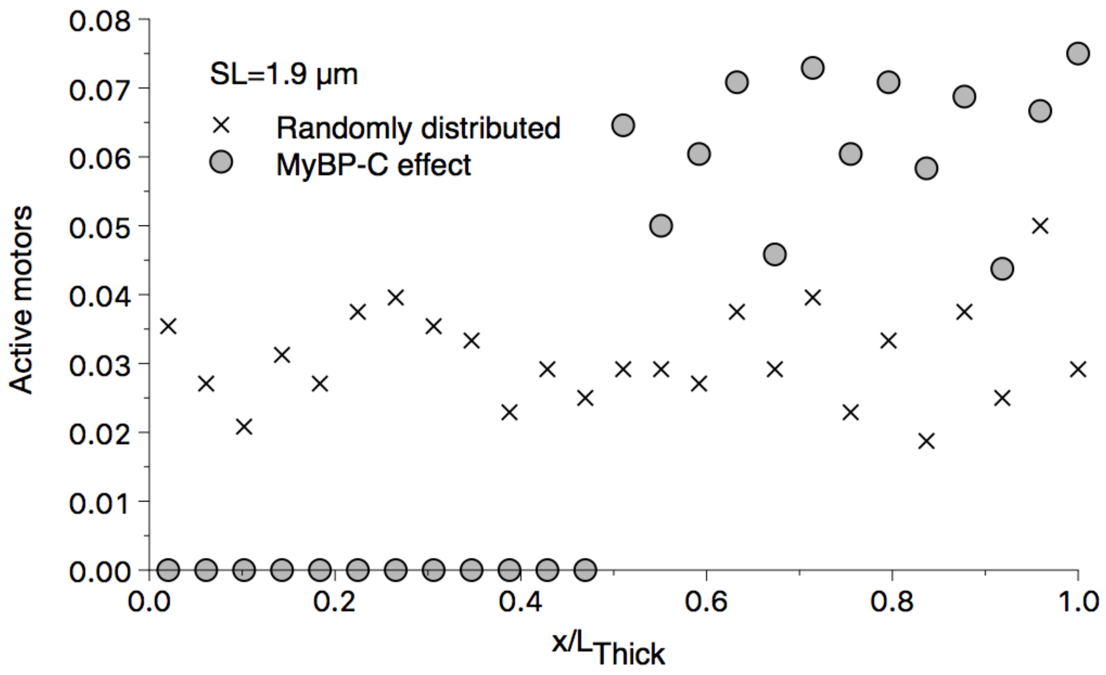

Figure S1: Percentages of active motors in the relaxed muscle with and without the effect of the MyBP-C. Under the simplification that myosin binding protein-C (MyBP-C) has no stabilizing effect in the myosin SRX state, a uniform and random distribution of the constitutively active motors is generated (crosses). To include the stabilizing effect, as an extreme case, a complete stabilization of the OFF state is assumed in the C-zone of the thick filament (about one half of the whole length starting from the M-line), while along the rest of the filament is imposed a uniform and random distribution (filled circles). In both cases, the whole amount of constitutively ON motors is the same (3%).

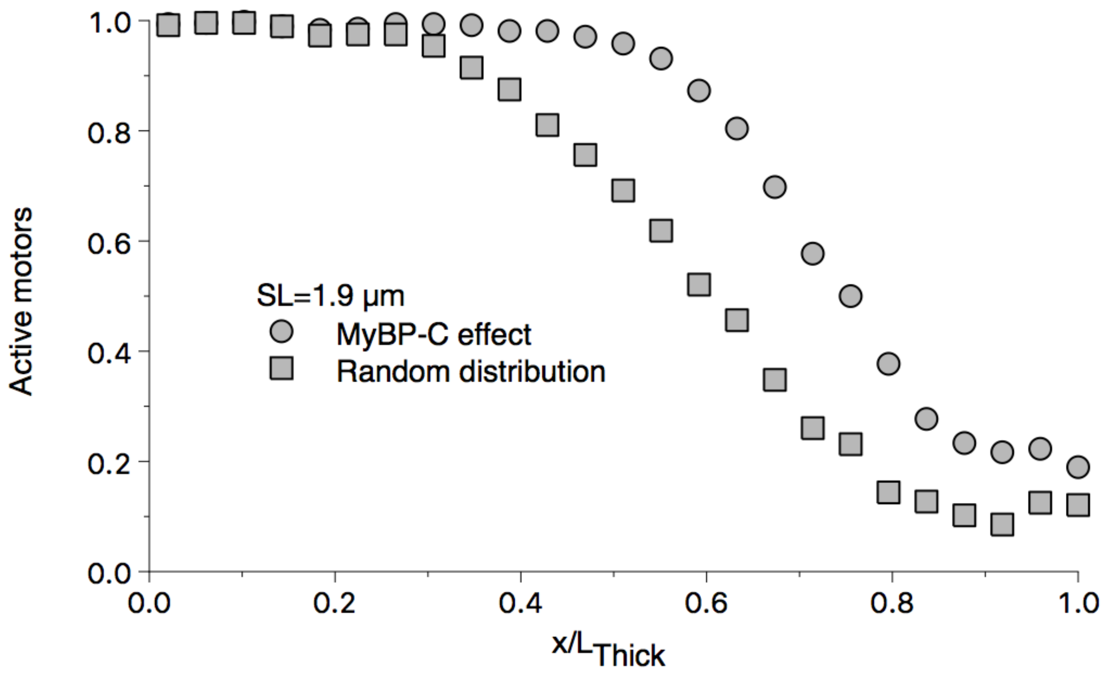

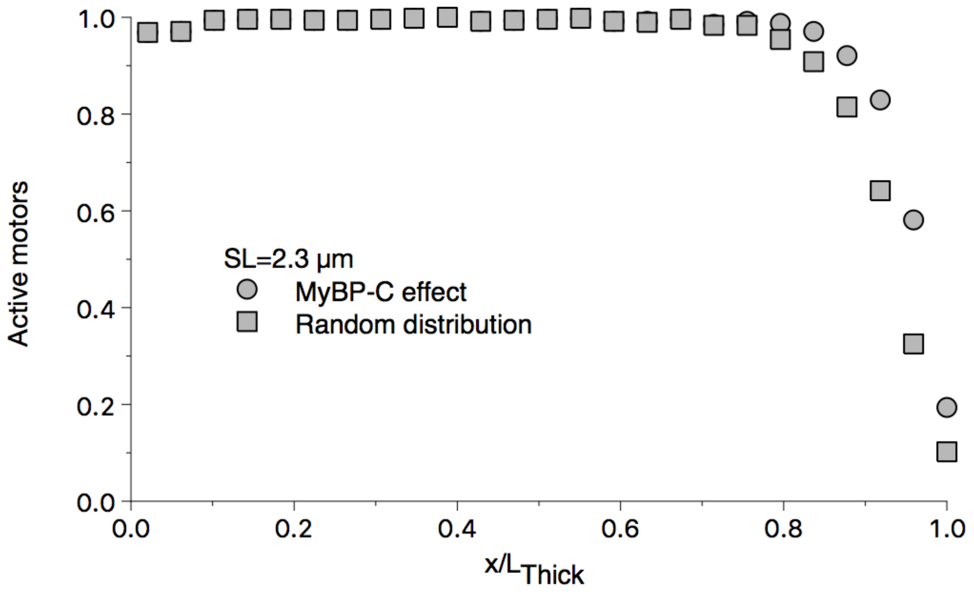

Figure S2: Effect of the different distributions of the constitutively active motors due to the presence of the MyBP-C, on the activated regions at high [Ca^{2+}]. At SL=1.9 μm (upper figure), the simulated effect of the MyBP-C (circles), leads to a wider fully activated region at high [Ca^{2+}], and, consequently, to a higher tension with the same parameters for the active cycle, respect the uniformly distributed constitutively active heads (squares). The effect is seen also at SL=2.3 μm (lower figure), even though the difference is smaller due to the wider fully activated region for the longer SLs.

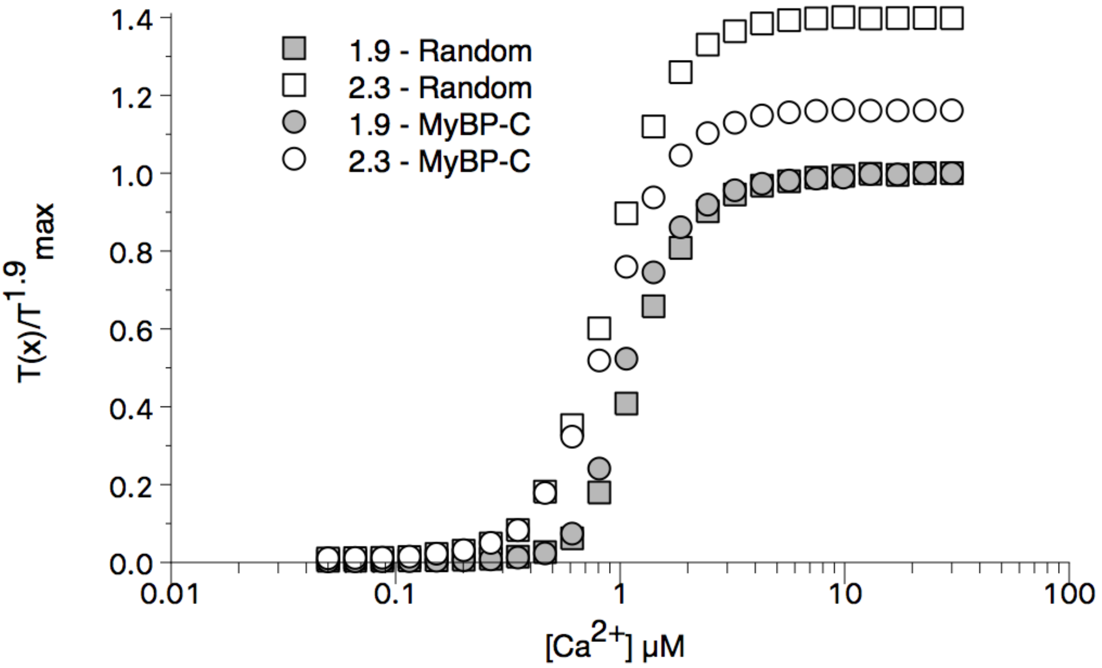

Figure S3: Normalized tension-[Ca^{2+}] at different SLs, in the presence or absence of the stabilizing effect of the MyBP-C. When normalized on the maximum tension generated at SL=1.9 µm, the increase of the tension at higher SLs is smaller when the constitutively active motors are concentrated toward the free end of the thick filament by the supposed stabilizing effect of the MyBP-C. This is due to the simulated smaller *reservoir*, related to the wider fully activated region.

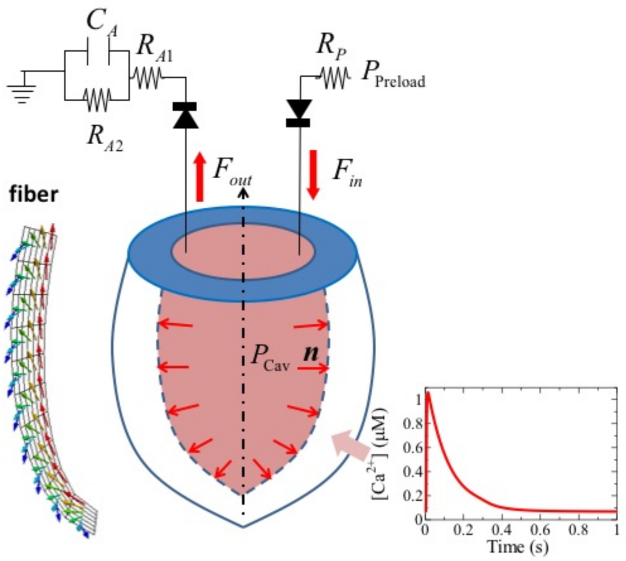

Figure S4: Finite element ventricle model. A rotationally symmetric model has been used for the FEM of the left ventricle. Lumped model for the peripheral circuit, fiber orientation and calcium transients are also shown and described in the text.
